# Clinical skills assessment: limitations to the introduction of an "OSCE" (Objective Structured Clinical Examination) in a traditional Brazilian medical school

**DOI:** 10.1590/S1516-31802004000100004

**Published:** 2004-01-08

**Authors:** de Almeida Troncon Luiz Ernesto

**Keywords:** Assessment, Clinical skills, University hospitals, Medical students, Undergraduate medical education, Estudantes de Medicina, Educação médica, Avaliação educacional, Ensino, Medicina, Faculdades de Medicina

## Abstract

**CONTEXT:**

Assessment of clinical skills has a central role in medical education and the selection of suitable methods is highly relevant. The OSCE (Objective Structured Clinical Examination) is now established as one of the most valid, reliable and effective tests for the assessment of clinical skills.

**OBJECTIVE:**

To describe student and faculty perceptions of an OSCE introduced in a traditional Brazilian medical school.

**TYPE OF STUDY:**

Descriptive, semi-quantitative study.

**SETTING:**

Faculdade de Medicina de Ribeirão Preto, Universidade de São Paulo.

**PARTICIPANTS:**

258 junior medical students finishing an introductory course on basic clinical skills and six faculty members deeply involved with the OSCE administration.

**PROCEDURES:**

Over a period of three consecutive years, student perceptions on the examination were evaluated using a structured questionnaire containing several five-point scales; faculty members' opinions were collected using a structured questionnaire plus a personal interview.

**MAIN MEASUREMENTS:**

Student satisfaction or dissatisfaction with aspects of OSCE administration and positive or negative opinions from faculty members.

**RESULTS:**

Students were comfortable with cases and tasks, but nearly half (48%) of them criticized organizational aspects of the OSCE. Substantial proportions of students reported difficulties with both time management (70%) and stress control (70%). Improvement of several aspects of exams reduced criticism of organization to a minority (5%) of students, but the proportions of students reporting difficulties with time management (40%) and stress control (75%) during the exam remained virtually unchanged. Faculty members acknowledged the accuracy of the OSCE, but criticized its limitations for assessing the integrated approach to patients and complained that the examination was remarkably time and effort-consuming. The educational impact of the OSCE was felt to be limited, since other faculty members did not respond to the communication of exam results.

**CONCLUSIONS:**

In addition to shortage of resources and organizational difficulties, local cultural aspects and the absence of a more favorable educational climate may hinder lasting improvements in assessment methods in traditional medical schools.

## RESUMO

**CONTEXTO:**

A avaliação das habilidades clínicas tem um papel central na educação médica, sendo importante o uso de métodos apropriados para esta finalidade. O exame clínico objetivo estruturado ("*OSCE*") constitui um dos mais válidos, fidedignos e efetivos meios de avaliação de habilidades clínicas.

**OBJETIVOS:**

Descrever as percepções de estudantes e professores frente à introdução de um exame "*OSCE"* em uma escola médica tradicional.

**TIPO DE ESTUDO:**

Estudo descritivo, semiquantitativo.

**PARTICIPANTES:**

Um total de 258 estudantes de Medicina que terminaram um curso sobre habilidades clínicas básicas e seis professores que se envolveram na administração da "*OSCE"*.

**PROCEDIMENTOS:**

Aplicouse aos estudantes um questionário estruturado para a medida da sua percepção sobre vários aspectos do exame. As opiniões dos professores foram colhidas com outro questionário, cuja aplicação foi seguida por uma entrevista pessoal.

**PRINCIPAIS MEDIDAS:**

Percentuais de estudantes satisfeitos ou insatisfeitos com os vários aspectos do exame e opiniões globais positivas ou negativas dos professores sobre a avaliação.

**RESULTADOS:**

Os estudantes mostraramse satisfeitos com os casos e as tarefas clínicas solicitadas, mas 48% deles criticaram aspectos organizacionais do exame. Proporções substanciais dos estudantes relataram dificuldades em controlar o tempo (70%) e o estresse emocional (70%) durante o exame. O aperfeiçoamento de vários aspectos do exame associouse à redução do percentual de estudantes insatisfeitos com a sua organização (5%), mas as proporções de estudantes relatando dificuldades com o controle do tempo (40%) e do estresse (75%) permaneceram inalteradas. Os professores reconheceram a acurácia do exame, mas criticaram a sua limitação em prover avaliação mais integral da abordagem do paciente. Houve, também, queixas de que o exame era consideravelmente dispendioso em termos do tempo e do esforço necessários para a sua aplicação. O impacto educacional do exame foi percebido como sendo limitado, visto que outros professores, não diretamente envolvidos com a sua administração, não mostraram interesse em discutir os seus resultados.

**CONCLUSÕES:**

Junto com a falta de recursos e a complexidade organizacional, particularidades de ordem cultural e a ausência de um ambiente educacional mais favorável podem dificultar o aperfeiçoamento dos métodos de avaliação nas escolas médicas tradicionais.

## INTRODUCTION

Assessment of clinical skills has a central role in medical education and the selection of suitable methods has been a matter of permanent concern for clinical teachers, course directors and medical educators.^1-3^ The OSCE (Objective Structured Clinical Examination)^4,5^ is now established as one of the most valid, reliable and effective tests for the assessment of clinical skills.^1,3^ In a typical OSCE, examinees rotate through a number of stations staffed by either real or standardized patients,6 where they are required to perform different clinical tasks. The examinees are observed and their performance is assessed using structured checklists.^5^

Although a considerable body of knowledge concerning technical and organizational aspects of OSCE administration is now available, little has been published on the responses of both student and faculty members of traditional medical schools to attempts at introducing OSCEs. In 1995, we introduced an OSCE with standardized patients for the summative assessment of junior medical students finishing an introductory course on basic clinical skills in a traditional Brazilian medical school. This was an isolated initiative by a small number of faculty members and, to the best of our knowledge, comprised the first successful application of an OSCE for the assessment of undergraduate medical students in the country.^7^ This OSCE was administered for three consecutive years, during which student and faculty member perceptions of the technique were recorded and used as feedback to improve the assessment. However, we found a number of limitations that precluded the permanent utilization of the OSCE. In this paper we report on the student and faculty member responses to this attempt, which highlighted some of the difficulties that may be found in the management of educational change.

## METHODS

### Settings

The Faculty of Medicine of Ribeirão Preto is located on an inland campus of the University of São Paulo in southeastern Brazil and is nationally regarded as one of the top medical schools in the country. This is largely due to the fact that faculty members are highly qualified and nearly all of them work full-time for the institution, including those from clinical departments. Although faculty members are highly committed to academic duties, the amount of individual time devoted to clinical, research and administrative work is in general higher than what is dedicated to teaching. Moreover, there are no regular programs for faculty development or specific training for educational activities. Although the undergraduate curriculum has been changed considerably since the early nineties,^8^ teaching is predominantly didactic and the assessment methods are focused mainly on cognitive aspects rather than in skills or competencies. The medical school receives annually 100 students aged 17-19 years, who enter shortly after finishing high school. The current local curriculum comprises two years of integrated basic sciences, one semester (third year) of preclinical disciplines and three semesters (third and fourth years) of clinical disciplines. The two final years (fifth and sixth years) are spent in internships in major clinical areas, such as Internal Medicine, General Surgery, Pediatrics and Gynecology & Obstetrics.

### Course structure

In the mornings of the preclinical fifth semester (third year), introductory courses for general and specialized areas of medicine are taught. In the Department of Medicine, an essentially practical course on basic clinical skills (communication and interaction with patients, history-taking and physical examination) is given. Sixteen groups of 6-7 students are assigned to clinical tutors, who typically demonstrate on real inpatients how a basic clinical skill should be performed, after which students work on the wards under the tutor's supervision, putting into practice the skills taught. Student assessment was based on both subjective tutor evaluation of student effort and summative exams, consisting mainly of long cases, which were not invariably observed by clinical tutors.

### OSCE design

The proposed OSCE comprised six stations, including two with simulated patients for the assessment of communication and interaction with patients, as well as for history-taking skills. The other four stations had real patients, with true signs, for the assessment of physical examination skills. This OSCE design was kept for three years and was not affected by a number of improvements that were added year by year in response to student and faculty member criticisms. Initially, the examination at each station lasted for five minutes and the degree of complexity of the required tasks was calibrated accordingly. For example, students were asked to briefly explore the patient's main habits and lifestyle characteristics at one of the simulated patient stations and perform focused chest percussion at one of the physical examination stations.^7^

Experienced faculty members observed and evaluated student performance with detailed checklists. At the four physical examination stations, small sets of "true/false" postencounter questions were used to assess the detection and the gross interpretation of relevant findings presented by the patient.

### Exam preparation and administration

From the first week of the semester until the examination day, during each of the three years of OSCE application, 6-10 faculty members were actively involved in exam preparation. From this group, six faculty members participated actively in all three OSCE runs. The examination was carried out in accordance with published recommendations^5,9,10^ and included the selection of contents and tasks, the recruitment and training of suitable people to act as simulated patients^11^ and the careful selection of stable patients, with clearly defined signs at the physical examination. Particular attention was paid to the elaboration of detailed checklists and to the definition of "pass/fail" criteria. Before the first administration, in 1995, a detailed blueprint was written and approved by the local Ethics Committee. Preparations also included arrangements related to space in an inpatient ward and assignment of nursing staff and administrative personnel.

In each of the three years of OSCE administration, the exams were held on two consecutive mornings, with 50 students being examined each morning. Every run demanded the work of eight faculty members for the entire morning period.

### Pass/Fail criteria

The checklists for the various stations contained eight to ten items corresponding to key skills. At any given station, a "pass" decision was taken for the student fulfilling at least 50% of the checklist items, a level that was set in order to conform to local regulations. Students failing in more than three stations were regarded as having failed the overall examination.

### Student perceptions

During the three years of OSCE administration, student perceptions of the exam were evaluated according to a grading system. From two to seven days after the exam students were asked to attribute a mark ranging from one ("very poor") to five ("excellent") to the following aspects of the OSCE: a) general organization of the exam; b) clinical vignettes and instructions for work at the stations; c) adequacy of the tasks required; d) quality of the post-encounter questions; and e) adequacy of the degree of complexity of the contents involved. Students were also asked to rank from one ("very low") to five ("very high"): f ) the difficulty in managing the time available at each station; and g) the degree of emotional stress elicited by the exam. Student dissatisfaction with any particular aspect of OSCE administration was characterized by attribution of lower grades (1 or 2) to the first five aspects, or higher grades (4 or 5) to the last two aspects evaluated. The questionnaire also contained a final item inviting students to comment openly about any aspect of the examination.

### Faculty opinions

After the last time the OSCE was administered, the six faculty members that had worked actively on the preparation and application of all three consecutive OSCE applications were asked to answer a structured questionnaire containing open questions related to the following aspects: a) general impression of the OSCE method; b) main perceived advantages and disadvantages of the examination; and c) influence of the OSCE results on teaching. The questionnaire also contained a final item inviting open remarks on any other aspect of the examination. After application of the questionnaire, all answers were further discussed and clarified in a personal interview. All opinions were categorized qualitatively as positive or negative according to their contents, without any concern about relevance ranking.

## RESULTS

### Student performance

In the first OSCE administration, 8% of students failed the examination. This percentage was slightly higher than the 3-5% fail rate historically recorded for the course. On the other hand, nearly 60% of students passed in at least five of the six OSCE stations. During the following two years, the fail rate fell to less than 5%, and the rate of students passing in at least five stations remained higher than 60%.

### Student perceptions and consequent modifications of the examination

The response rates to the questionnaires throughout the three years were greater than 90%, thus representing a total of 258 students.

Student opinions expressed throughout the three years of OSCE administration are summarized in Table 1. Student dissatisfaction was generally low regarding clinical vignettes, tasks required, post-encounter questions and contents involved. On the other hand, during the first year, 48% of students were dissatisfied with exam organization (Table 1), and 70% of respondents were discontent with the time available at each station and attributed higher grades to the degree of emotional stress elicited by the examination.

**Table 1:** Student perception of an "OSCE" examination for the assessment of basic clinical skills. Percentages of students dissatisfied with the various aspects of the examination, according to the answers to a structured questionnaire applied over three consecutive years following OSCE introduction in Brazilian medical school

Aspects evaluated	Year of OSCE administration		
	1st (n = 82)	2nd (n = 86)	3rd (n = 90)
General exam organization	48	15	5
Clinical vignettes and instructions	15	10	5
Adequacy of the tasks required	10	5	15
Quality of post-encounter questions	8	7	8
Complexity of the content involved	15	12	10
Difficulty with time management	70	55	40
Emotional stress	70	69	75

*OSCE = Objective Structured Clinical Examination; n = number of students answering the questionnaire.*

In the open part of the questionnaire, many students added remarks that were relevant to the above mentioned aspects. There were also comments about the premises being too small and that there were too many post-encounter questions, which contributed to a perception that the time for the work was too short. A large number of students complained that the presence of an observer at all the stations was intimidating, which contributed to a feeling that the examination was exceedingly stressful.

In order to solve the perceived problems, a number of measures were taken for the second year of OSCE administration, which were also kept for the following year. These measures included an effort at marketing the examination, with periodic communication of general information on the examination characteristics and structure to students during the semester. Also, a more extensive and careful briefing was carried out in the days preceding the exams. Arrangements were made to assure that the exam was held in larger and more comfortable wards, with improved conditions in relation to auxiliary personnel and organization. The length of time spent at each station was increased to six minutes and both the required tasks and questions were simplified. Also, two "rest" stations were inserted among the six stations, filled only with cartoon strips for student relaxation. Moreover, the simulated patients were intensively trained to both portray the exam cases and to assess student performance, so that an observer was no longer needed in the 2 stations concerning communication and history-taking skills.

During the following two years, the proportions of students who were critical of exam organization fell to 15% and then to 5% (Table 1). However, difficulties with time control were still reported by at least 40% of the examinees (Table 1) and there was no change in the percentages of students regarding the exam as highly stressful, since in the second and third years of OSCE administration, 69% and 75% of students, respectively, attributed scores of higher than three to the degree of emotional stress (Table 1).

### Faculty member perceptions

The opinions expressed by faculty members deeply involved with OSCE administration are summarized in Table 2. All faculty members agreed that the OSCE was more relevant and accurate than previous examinations and particularly acknowledged the aspects relating to objectivity and standardization. It was also mentioned by all faculty members that the OSCE had probably been very useful in showing students what skills are important. The OSCE results were also seen as helpful for this group of clinical tutors in relation to teaching more uniformly. Moreover, the OSCE was perceived as highly effective in revealing a detailed picture of both student performance and course efficacy.

**Table 2:** Main faculty members opinions about an "OSCE" examination for the assessment of basic clinical skills of medical students in a Brazilian school. Opinions were collected using a structured questionnaire and a subsequent interview and categorized as positive or negative accordingly to their content

**POSITIVE**
• The technique is highly relevant and more accurate than previous examination methods.
• The examination may be useful for transmitting to students what skills are important to learn.
• The method is effective for assessing what is taught by different tutors and how this is learned.
• The examination may help to achieve more uniform teaching
**NEGATIVE**
• This method is unable to provide assessment of the integrated approach to patients.
• OSCE = Objective Structured Clinical Examination

*OSCE = Objective Structured Clinical Examination*

Since the faculty members involved in the OSCE were also clinical tutors, it was not surprising that all of them reported a perception of positive influence on their personal way of teaching. However, it was also pointed out that it had not been feasible to exchange experiences with the remaining tutors that were not directly involved in the examination, due to the lack of time and interest on the part of the others.

Faculty members reported that the OSCE possibly had a positive effect on students' drive to actually study and practice. This effect was felt to be greater than what could be associated with the previous examination method. On the other hand, all but one faculty member criticized the limitation of the OSCE for assessing the integrated approach to patients. It was emphasized that this aspect might represent a dissociation between the examination and the objectives of the main course. One responder even expressed the concern that the exam, which was designed as a system-oriented series of stations, might actually transmit to junior students a distorted view of "the sick person as merely an array of organs with disorders". Faculty members also questioned the validity of examining highly stressed students and regretted the lack of facilities for both getting students more used to the exam technique and providing immediate feedback.

Finally, the OSCE was regarded as being remarkably demanding in terms of faculty time and effort, particularly by those more directly involved in the selection and preparation of real patients, and the recruitment and training of people to act as simulated patients.

### OSCE replacement

By the end of the third year of OSCE administration, clinical tutors and the course director had agreed that keeping this model of clinical skills assessment would be worth-while only if the negative aspects expressed in both faculty member and student responses could be overcome. However, acknowledging the present-day difficulties in further improving the exam, it was then decided to replace this summative OSCE with assessment events that were more formative, iterative and smaller, even though less standardized.

## DISCUSSION

The experience of introducing an OSCE at a rather conservative medical school without a tradition of objective examinations of students' clinical skills provided an opportunity to learn about student and faculty member responses. Reflection on this experience has also raised a number of issues related to general aspects of medical education and assessment, as well as the management of educational change.

Data on students' perceptions about the examination yielded important information that was helpful for improving the organization of the OSCE. Nevertheless, none of the proposed modifications was able to reduce the relatively high levels of emotional stress felt by our students during the examination. Exams are a well-known source of stress and OSCEs in particular are regarded as quite stressful,^12^ although it has been suggested elsewhere that an OSCE is less stressful to students than previous examination experiences.^10^ It is unlikely that stress in our students was caused by unfamiliar tasks or content included in the various stations, because students seemed to be comfortable with these aspects of the examination from the time of the first OSCE administration. On the other hand, it is conceivable that student stress could be related to fears concerning possible failure, not-withstanding the fact that the observed rates of student failure were similar to those expected for the course. Student stress could also originate from local cultural factors, since students in this country tend to perceive assessment procedures and tests as something aiming only at "rewarding a few students and punishing others".^13^ Therefore, the introduction of a new examination may have been seen as a rather threatening experience.

The difficulties on the part of students in managing time during the work at the OSCE stations could not be ascribed to excessively short lengths of time at stations, since the time at each station was similar to what has been recommended for the assessment of very basic clinical skills.^9,14^ This kind of difficulty persisted even with longer times at stations and simpler tasks and a smaller number of post-encounter questions, and thus might be related to different factors, including student immaturity and lack of specific training in time management skills. Lack of practice at being examined in the OSCE format might also have contributed to both the dissatisfaction with the time available and the perceptions of the OSCE as a highly stressful examination. These negative feelings might have been minimized by prior administration of one or two mock ("*dry run"*) examinations. However, this was thought to be unfeasible because of number of factors, including lack of time in the curriculum grid, shortage of space in a very busy general hospital and unavailability of faculty members to prepare the examination.

The small number of faculty members involved in exam preparation and application seemed to be convinced that the proposed OSCE was more accurate than traditional methods previously utilized. Indeed, OSCEs are regarded as a highly valid and reliable method for the assessment of clinical skills.^1-3^ This is attested not only by the worldwide spread of this examination model throughout medical schools10,12 and residency programs,^15-17^ but also by its use in high-stake examinations such as those carried out by the Medical Council of Canada^18^ and the ECFMG (Educational Commission for Foreign Medical Graduates) of the United States.^19^

Nevertheless, some faculty members expressed the concern that the OSCE applied in our department was insufficient for assessing the integrated approach to patients, and thus might represent a dissociation between the general objectives of the undergraduate curriculum and the assessment strategy. This sort of discrepancy involving OSCE-based assessment of clinical skills had already been pointed out previously.^20^ A more comprehensive approach could have been facilitated by designing longer times spent at each station, with the same sequence of tasks normally involved in real clinical encounters (history-tak- ing followed by physical examination and clinical reasoning), as in the Clinical Perform- ance Exercise^21^ or in the ECFMG Clinical Skills Assessment.^19^ However, again, this would be unfeasible given the shortage of time and space available. On the other hand, the tasks and content included in the exam, involving focused history-taking and physical examination of cases that present with common clinical conditions,^7^ were similar to the original OSCE description^4^ and, moreover, consistent with the more specific course objectives, as an introduction. Accordingly, these aspects of the examination were well accepted by the examinees.

Our faculty members also acknowledged the high educational value of the OSCE, as feedback information that may potentially lead to improvements in both student learning^22^ and clinical teaching.^12^ However, this was limited in our case, since giving immediate feedback to the students was not possible due to the practical reasons already mentioned, particularly the limited number of mornings available for the assessment. It could be argued that feedback to students could be delivered using other means, such as group discussion on the days following the examination, or by posting commented individual results. Again, this was felt to be unattainable on that occasion, due to lack of time in the curriculum grid as well as the shortage of both faculty member time and secretarial assistance. Nevertheless, further refinements of exam application in our institution allowed immediate feedback to students to be delivered, and proved that this resource is associated with improved student satisfaction about being examined (author's personal observations).

The impact of the OSCE results on clinical teaching was also perceived to be limited, since faculty members not directly involved in preparing and running the exams did not respond at all to written communication of the results, nor were they able to attend meetings for discussion of the educational meaning of the observed results. This was ascribed to lack of time and interest on the part of faculty members deeply involved in clinical work and research activities, which may reflect a shortage of effective mechanisms for rewarding teaching as much as other clinical and academic activities.^23,24^ The scarcity of such mechanisms for rewarding faculty member engagement in educational activities, such as the organization and administration of more elaborate examinations, may also have contributed towards building the perception that planning and running an OSCE is excessively time and effort-consuming, which played a key role in the decision to replacing this assessment model with a less complex examination.

A more favorable attitude towards assessment and educational innovations among faculty members might be expected if our institution were to have a regular faculty development program. This would theoretically help faculty members to acknowledge the benefits of a more valid and reliable method for student assessment and favor the fostering of an "evaluation culture".^25^ Indeed, the Brazilian system of higher education is regarded as lacking in such culture, since many teachers tend to perceive assessment procedures only as a bureaucratic means of deciding who, among the student population, is going to pass or fail.^13,25^

Absence of favorable external factors may also have contributed to the lack of a more positive attitude among faculty members towards assessment of students' clinical competence. Medical schools in this country are accredited and regulated by the Ministry of Education, which only very recently set a number of general recommendations concerning the objectives of undergraduate medical education.^26^ These recommendations include an explicit list of core competencies that a medical graduate should develop, but definitions of predetermined standards for clinical skills performance are still lacking, and the recommendations concerning assessment are rather vague.^26^ Furthermore, there is no explicit set of requirements for the accreditation of medical schools, and the performing of formal assessments of students' clinical competence using accurate methods does not seem to have a substantial weight in the awarding of reaccreditation to any given medical school. Therefore, performing costly and laborious examinations of clinical skills, although valid and reliable, does not yet seem to make so much sense for many teachers and course directors in this country.

The experience herein reported of introducing an OSCE in a traditional medical school highlights some aspects of the management of change in a rather conservative educational climate. Substantial changes in assessment have previously been reported else- where in the context of major curriculum reform, in countries where the educational climate seems to be more favorable.^27-30^ These experiences almost invariably involved the entire medical school and were conducted under powerful leadership, with access to adequate funding. In contrast, the present initiative originated from a small number of faculty members without any specific budget, but with concerns about the low accuracy of student assessment in an isolated course. Also, the proposed small-scale change in the assessment model did not represent a response to student or faculty dissatisfaction, nor was it implemented to comply with recommendations from authorities or institutions controlling undergraduate medical education.^31^ It is therefore plausible that the absence of stronger internal and external forces favoring educational improvement, together with the limited aspect of the change, may also have contributed to the loss of the proposed OSCE.

## CONCLUSIONS

The introduction of an OSCE into a traditional Brazilian medical school taught us about the variety of factors that limited the utilization of more objective, standardized methods for examining clinical skills and gave rise to difficulties in the management of changes in assessment methods. These factors not only included practical aspects, especially in relation to shortages of both human and material resources and organizational difficulties, but also local cultural factors and circumstances that caused low student and faculty acceptance. Particularly, the absence of a more favorable educational climate was felt to be an important limitation that precluded the permanent utilization of the proposed examination.
